# Supplementary material for: Complementary structural and functional abnormalities to localise epileptogenic tissue
Source: eBioMedicine. 2023 Oct 27;97:104848. doi: 10.1016/j.ebiom.2023.104848 (PMC10630610; doi:10.1016/j.ebiom.2023.104848)
Supplement: Supplementary Methods S1 and S2, Figs. S1–S14, and Analysis S1–S7 [file mmc1.docx]

**Supplementary Contents**

**Supplementary Methods 1 - Full Description of dMRI Acquisition and Processing**

**Supplementary Methods 2 - Full Description of iEEG Acquisition and Processing**

**Supplementary Analysis 1 - Correlation between dMRI and iEEG Abnormalities**

**Supplementary Analysis 2 - Decision Tree Prediction using LOOCV**

**Supplementary Analysis 3 - Robustness of Results to Different Resection Thresholds**

**Supplementary Analysis 4 - Alternative Case Studies**

**Supplementary Analysis 5 - Resection of Seizure Onset**

**Supplementary Analysis 6 - MRI-positive and MRI-negative Subgroup Analysis**

**Supplementary Analysis 7 - Further Example Patients (TLE and eTLE)**

**Supplementary Methods 1 - Full Description of dMRI Acquisition and Processing**

The following describes the dMRI acquisition and processing in full, as detailed in a previous paper (Horsley et al., 2022):

For the first cohort (39 patients and 29 controls), scans were collected between 2009 and 2013. For this cohort, MRI studies were performed on a 3T GE Signa HDx scanner (General Electric, Waukesha, Milwaukee, WI). Standard imaging gradients with a maximum strength of 40 mTm-1 and slew rate 150 Tm-1s-1 were used. All data were acquired using a body coil for transmission, and 8-channel phased array coil for reception. Diffusion-weighted MRI data were acquired using a cardiac triggered single-shot spin-echo planar imaging sequence (Wheeler-Kingshott et al., 2002) with echo time = 73 ms. Sets of 60 contiguous 2.4 mm-thick axial slices were obtained covering the whole brain, with diffusion sensitizing gradients applied in each of 52 non-collinear directions (b value of 1,200 s/mm2 [δ = 21 ms, Δ = 29 ms, using full gradient strength of 40 mTm-1]) along with 6 non-diffusion-weighted scans. The gradient directions were calculated and ordered as described elsewhere (Cook et al., 2007). The field of view was 24 cm, and the acquisition matrix size was 96 × 96, zero filled to 128 × 128 during reconstruction, giving a reconstructed voxel size of 1.875 × 1.875 × 2.4 mm. The dMRI acquisition time for a total of 3480 image slices was approximately 25 min (depending on subject heart rate).

For the second cohort (4 patients and 67 controls), scans were collected between 2014 and 2019. For this cohort, MRI studies were performed on a 3T GE MR750 scanner. Standard imaging gradients with a maximum strength of 50 mTm-1 and slew rate 200 Tm1s-1 were used. All data were acquired using a body coil for transmission, and 32-channel phased array coil for reception. Diffusion-weighted MRI data were acquired using a single-shot spin echo planar imaging sequence with echo time = 74.1 ms. Sets of 70 contiguous 2 mm-thick axial slices were obtained covering the whole brain. A total of 115 volumes were acquired with 11, 8, 32, and 64 gradient directions at b-values of 0, 300, 700, and 2500 s/mm2 respectively (δ = 21.5 ms, Δ = 35.9 ms) as well as a single b = 0-image with reverse phase-encoding (B0). The field of view was 25.6 cm, and the acquisition matrix size was 128 × 128, giving a reconstructed voxel size of 2 × 2 × 2 mm.

Diffusion-weighted MRI data were first corrected for signal drift (Vos et al., 2017), then eddy current and movement artefacts were corrected using the FSL eddy_correct tool (Andersson and Sotiropoulos, 2016) (first cohort) or using EDDY/TOPUP (second cohort). The b vectors were then rotated appropriately using the ‘fdt-rotate-bvecs’ tool as part of FSL (Jenkinson et al., 2012; Leemans and Jones, 2009). The diffusion data were reconstructed in MNI-152 space using q-space diffeomorphic reconstruction (QSDR) (Yeh and Tseng, 2011) with a diffusion sampling length ratio of 1.2. The HCP-1065 tractography atlas (Yeh et al., 2018) was used to determine connections between regions. The use of a tractography atlas is expected to result in fewer false positive connections than fibre tracking algorithms, since each tract has been visually confirmed to be expected and not spurious. This approach has the benefit of reducing the influence of network density on the subsequent group comparisons of networks (van Wijk et al., 2010), and has been used previously (Sinha et al., 2021; Moreira da Silva et al., 2020). A connection between MNI-152 space regions of the same parcellation was defined as present if streamlines passed into both regions in the corresponding region pair.

**Supplementary Methods 2 - Full Description of iEEG Acquisition and Processing**

The following describes the iEEG acquisition and processing in full, as detailed in a previous paper (Taylor et al., 2022). The subjects used in the present study are a subset of those described below:

In order to establish a standard reference for the spectral characteristics of intracranial EEG (iEEG), we utilized the RAM dataset. Channels labeled as seizure onset zone, early propagation zone, brain lesions, or having bad contacts were excluded. For each participant, we selected a 70-second segment of iEEG recording obtained during a state of relaxed wakefulness, shortly before engaging in a memory task. The remaining EEG signals from the remaining channels underwent visual inspection to identify recording artifacts, and channels located in white matter were also eliminated. This process resulted in a final set of 21,598 channels across 234 participants.

To compare and evaluate against the established normative baseline, we utilized a separate iEEG dataset from UCLH. Similarly, we retrospectively extracted a 70-second segment of interictal iEEG recording for each participant, ensuring that it was at least 2 hours distant from any seizure activity. Whenever possible, the recordings were obtained around 2 p.m. to increase the probability of wakefulness. Due to the retrospective nature of the study, the exact brain state during the recordings could not be determined.

In the UCLH dataset, we included all channels within the grey matter, including those within the seizure onset zone, propagation zone, and irritative zones. We only excluded channels with artifacts and those located in white matter, resulting in a total of 4,256 channels across 62 patients.

Prior to creating the normative map, all EEGs from the RAM dataset were downsampled to a sampling frequency of 200 Hz. In the UCLH dataset, various sampling frequencies were used: 256 Hz for two participants, 512 Hz for 52 participants, 1024 Hz for eight participants, and 2048 Hz for one participant. After applying a common average reference to all recordings in all participants, we estimated the power spectral density using Welch's method, employing a window size of 2 seconds with a 1-second overlap and a Hamming window function, for each 70-second recording.

The average power within five frequency bands of interest (delta: 1-4 Hz, theta: 4-8 Hz, alpha: 8-13 Hz, beta: 13-30 Hz, and gamma: 30-80 Hz) was then calculated using MATLAB's 'bandpower' function. To avoid power line artifacts in both the US and UK recordings, data between 47.5 and 52.5 Hz, as well as 57.5 and 62.5 Hz, were excluded specifically from the gamma band. The resulting band power estimates were then logarithmically transformed (base 10) and normalized to ensure that they summed to one for each contact, using the L1 norm. These transformed and normalized values represent the relative band power used throughout the study's results.

For the UCLH dataset, the clinical team also provided information on whether any channels exhibited interictal spikes at any point during the recording. This additional information was later used as a baseline measure and to further demonstrate the reliability and validity of our findings.

**Supplementary Analysis 1 - Correlation between dMRI and iEEG Abnormalities**

We investigated whether there was a correlation between dMRI connectivity abnormalities and iEEG band power abnormalities within individual patients. In the main text (Figure 3), we presented two example patients. In these patients, the abnormalities are correlated in one patient (Patient 1), but not in the other (Patient 2). These patients are presented below in Supplementary Figure 1 Panel A.

We computed the Pearson correlation between abnormalities for each patient. This is shown in Panel B. Across the cohort, the average correlation was not positively (or negatively) skewed (p=0.83). We tested this using a Wilcoxon signed rank test.


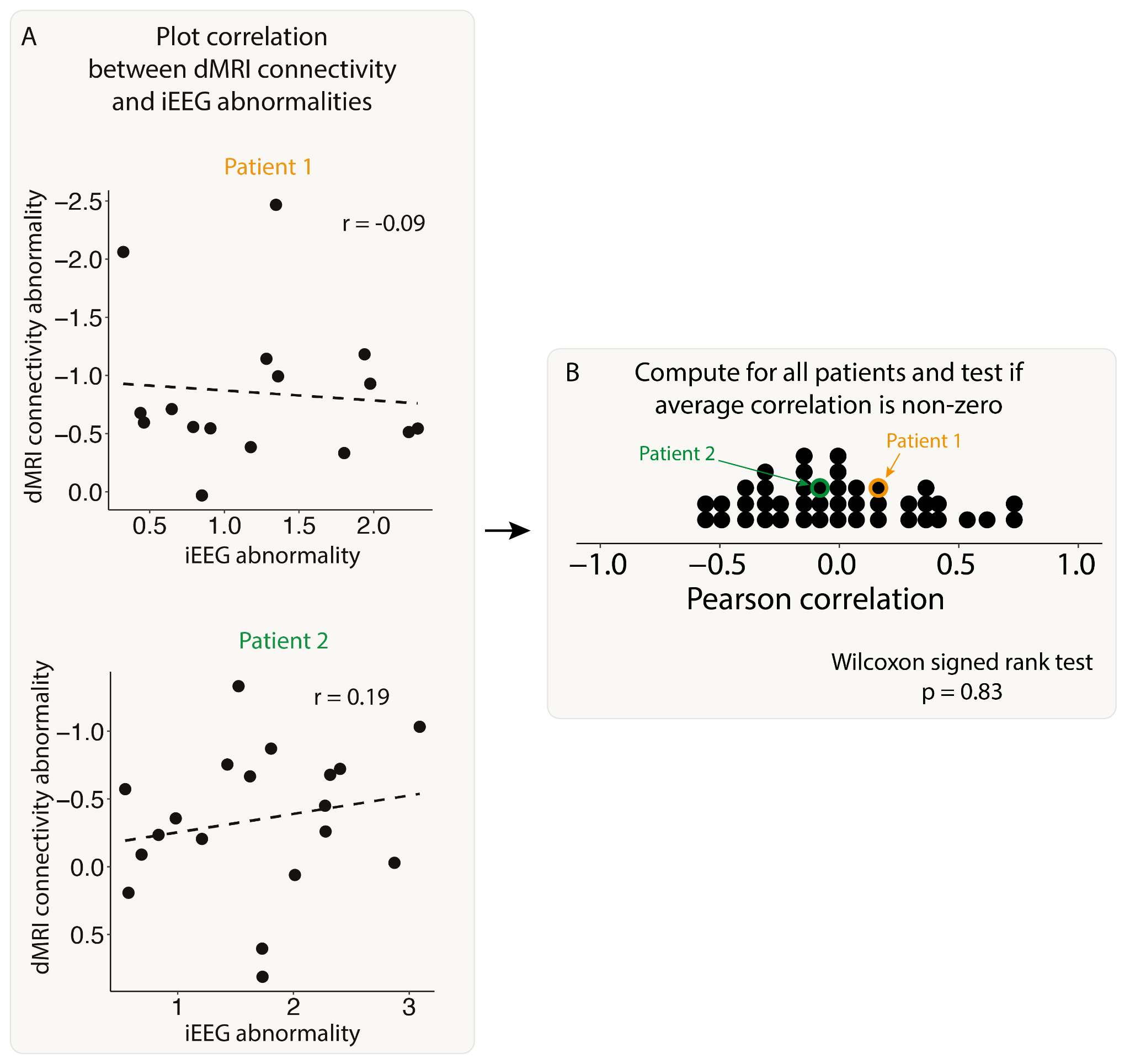


**Supplementary Figure 1: Across the cohort, there was no significant correlation between dMRI connectivity abnormalities and iEEG abnormalities.** A) Two example patients are presented with the Pearson correlation between abnormalities. B) Across the cohort, the average correlation was not significantly non-zero (p=0.83).

**Supplementary Analysis 2 - Decision Tree Prediction using LOOCV**

In the main text, we presented the classification accuracy for seizure-free vs not-seizure-free patients using a decision tree. This provided an indication of the separability of the two classes (SF and NSF) using two D_RS_ measures, in a similar way to a ROC AUC statistic. However, machine learning methods such as decision trees are usually applied using cross-validation to give an estimate of how a model might perform on new, unseen data.

In a supplementary analysis to the main text, we used leave-one-out cross validation to predict the outcome of unseen patients using a decision tree. In summary, this removed one patient from the training set, fit a hypothesis-driven decision tree using connectivity D_RS_ and iEEG D_RS_ values on the remaining 42 patients, before predicting the excluded patients seizure freedom class (SF or NSF). We specified that the tree had one cut using connectivity D_RS_ and one cut using iEEG D_RS_ values. This process was repeated for all patients.

The decision tree (using LOOCV) predicted unseen patients with an accuracy of 72% (sensitivity = 0.76, specificity = 0.67). For each patient, similar cuts were used. These are shown in Supplementary Figure 2.


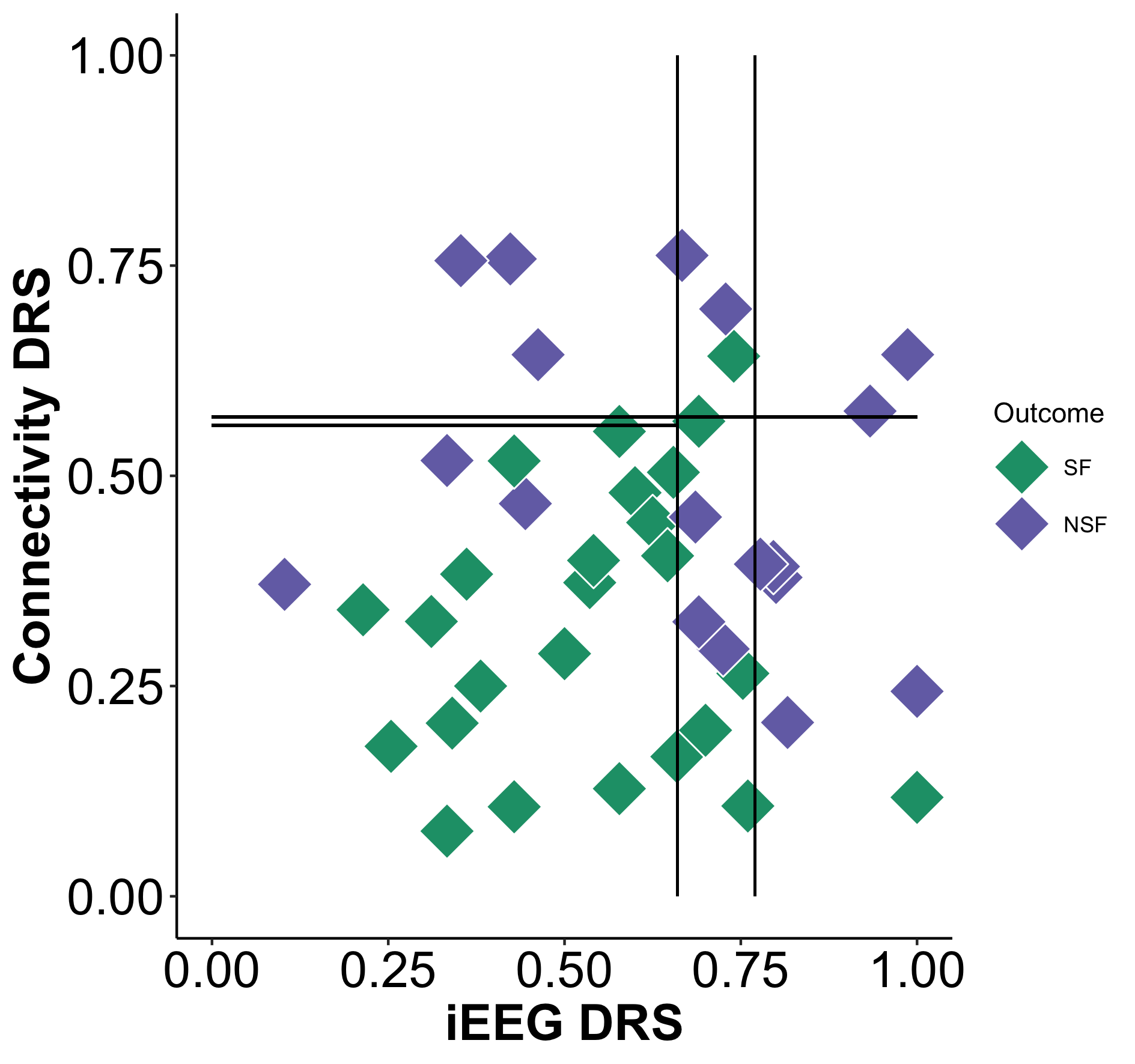


**Supplementary Figure 2: Separation of seizure-free vs not seizure-free patients using a decision tree with leave-one-out-cross-validation.** The straight lines on the plot show the locations of the decision-tree cuts for each individual model fit (43 individual models fitted).

**Supplementary Analysis 3 - Robustness of Results to Different Resection Thresholds**

In the main manuscript, we presented results with a resection threshold of 10%. Here, we examine the robustness of this threshold by re-running the analysis at thresholds of 25% and 50%.

**Resection threshold = 25%**

In a replication of the SVM analysis, fewer patients could be successfully separated as having distinct resected and spared zones. For those that were successfully separated, similar results were observed. Patients with maximal abnormalities resected were more likely to be seizure free (accuracy = 0.76, sensitivity = 0.71, specificity = 0.88, p = 0.02). For patients who could not be separated, seizure free outcome was more likely (13 SF vs 4 NSF, p=0.05).


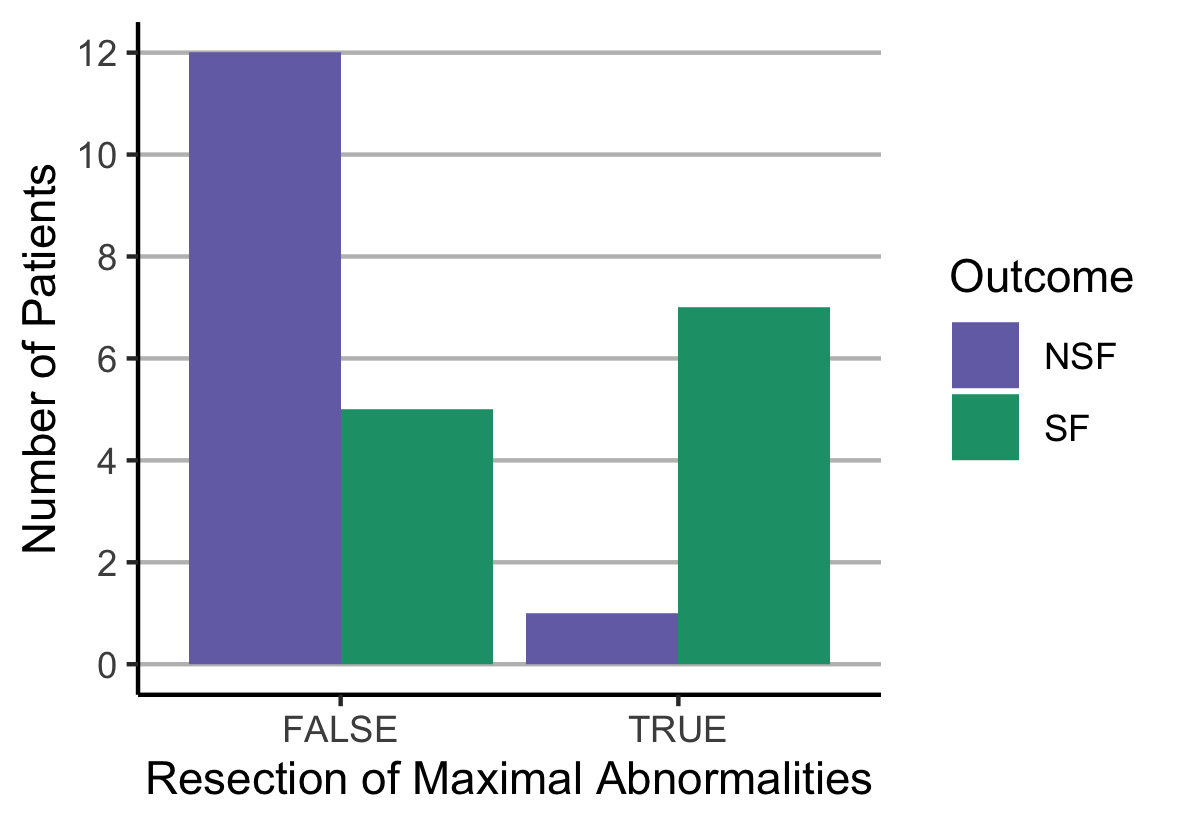


**Supplementary Figure 3: Resection of maximal abnormalities vs post-surgical outcome, at a regional resection threshold of 25%.**

In a replication of the iEEG D_RS_ analysis, similar results were observed. Patients with poor post-surgical outcomes were significantly more likely to have larger iEEG D_RS_ values (AUC = 0.72, p = 0.008).

In a replication of the connectivity D_RS_ analysis, similar results were observed. Patients with poor post-surgical outcomes were significantly more likely to have larger connectivity D_RS_ values (AUC = 0.73, p = 0.006).


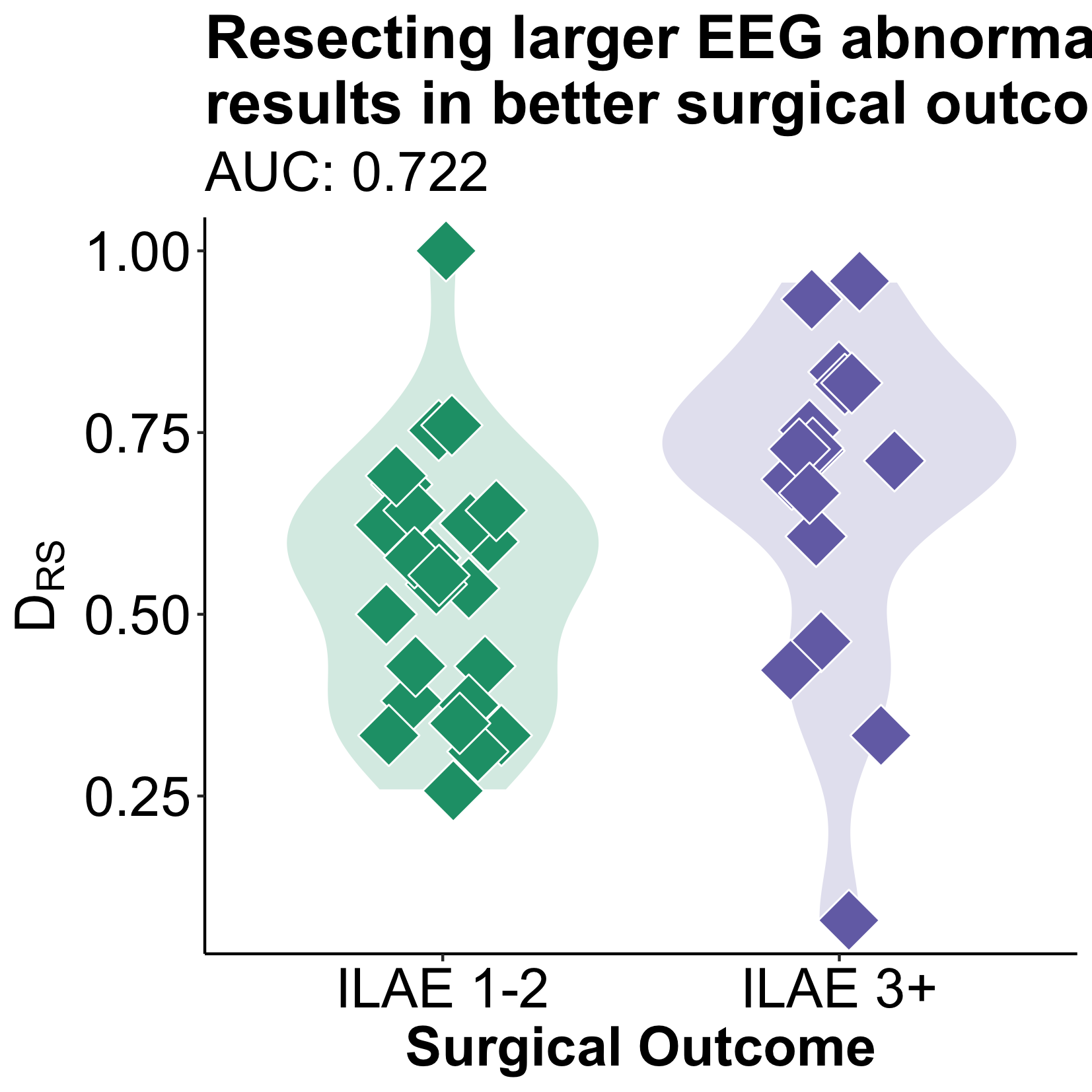

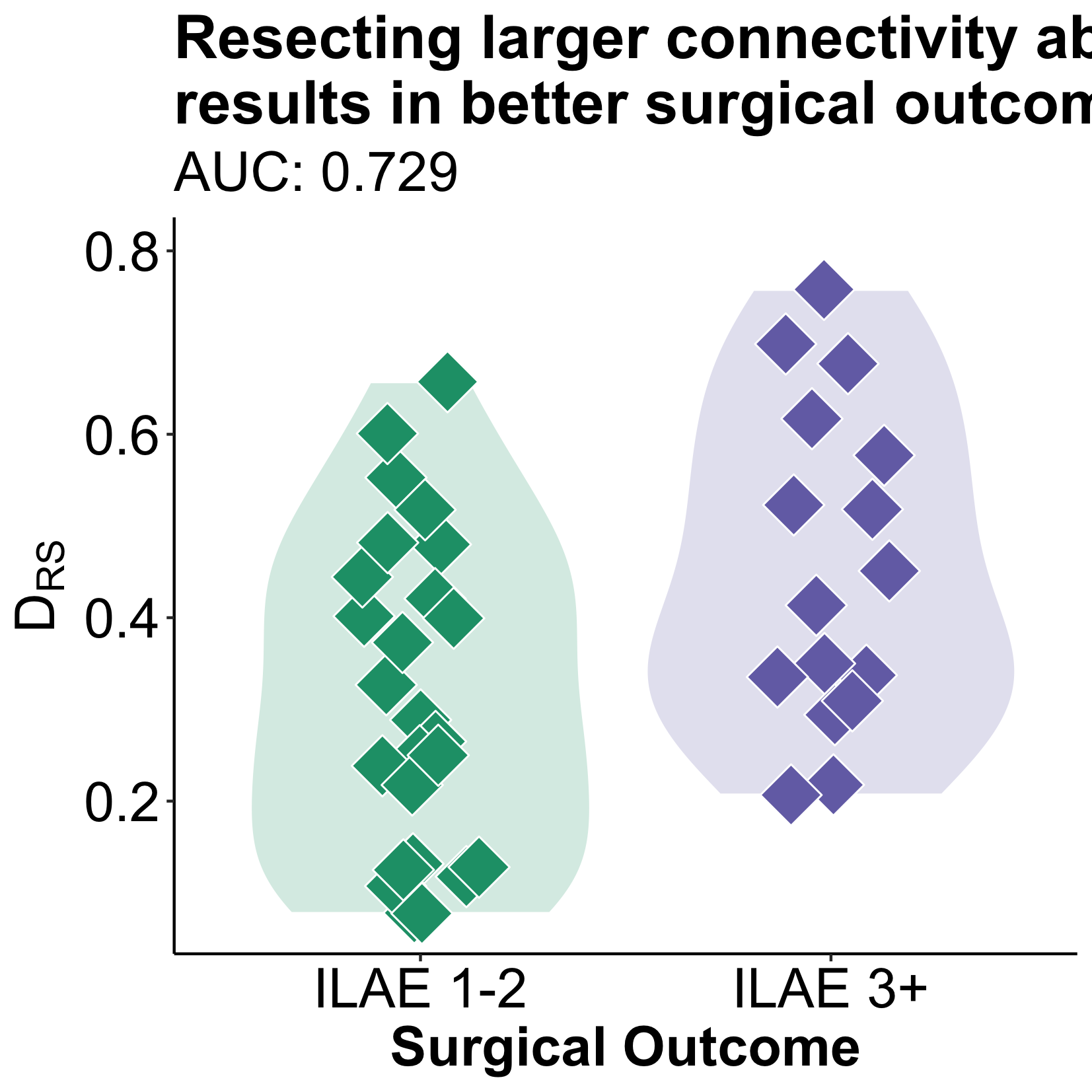


**Supplementary Figure 4: Patients with poor post-surgical outcomes had greater iEEG D_RS_ values (left) and larger connectivity D_RS_ values (right), at a resection threshold of 25%.**

**Resection threshold = 50%**

In a replication of the SVM analysis, fewer patients could be successfully separated as having distinct resected and spared zones. For those that were successfully separated, similar results were observed. Patients with maximal abnormalities resected were more likely to be seizure free (accuracy = 0.71, sensitivity = 0.62, specificity = 1.00, p = 0.11). For patients who could not be separated, neither seizure free nor not-seizure free outcomes were more likely (p=0.38).


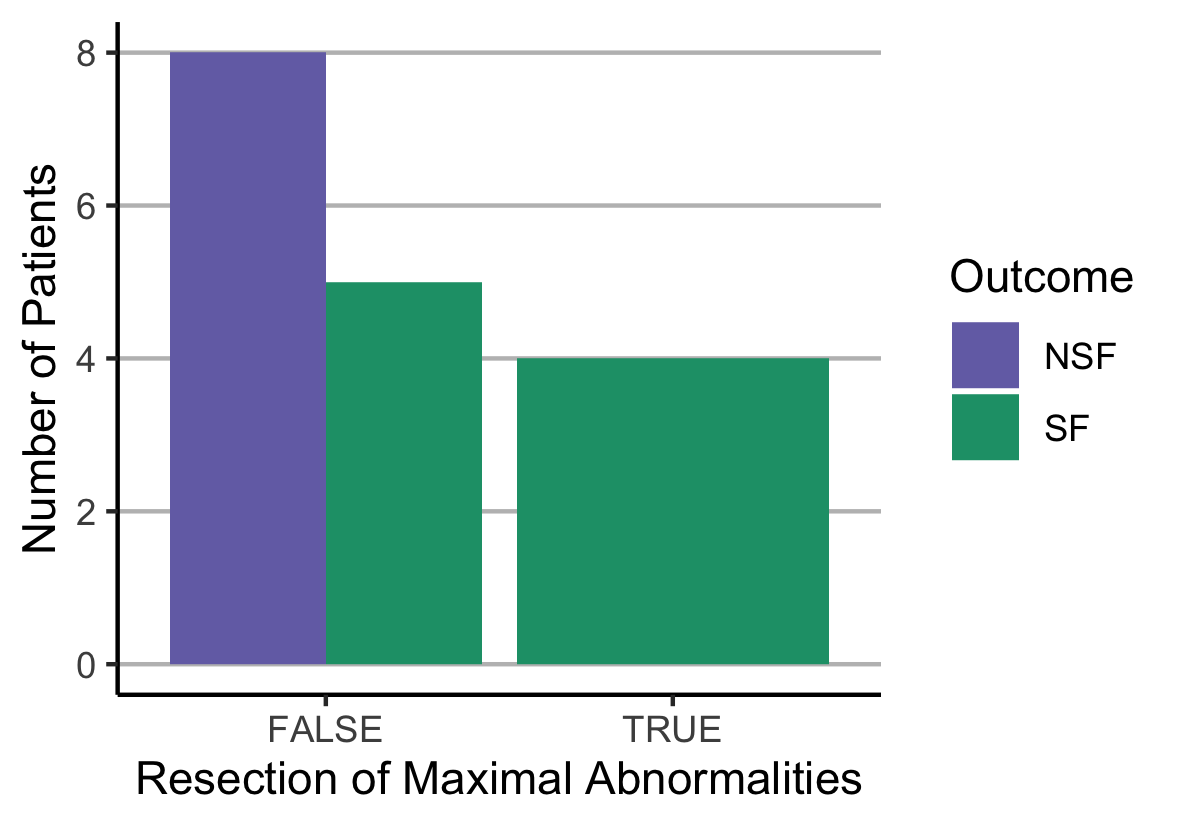


**Supplementary Figure 5: Resection of maximal abnormalities vs post-surgical outcome, at a regional resection threshold of 50%.**

In a replication of the iEEG D_RS_ analysis, similar results were observed. Patients with poor post-surgical outcomes were significantly more likely to have larger iEEG D_RS_ values (AUC = 0.78, p = 0.002).

In a replication of the connectivity D_RS_ analysis, similar results were observed. Patients with poor post-surgical outcomes were significantly more likely to have larger connectivity D_RS_ values (AUC = 0.69, p = 0.02).


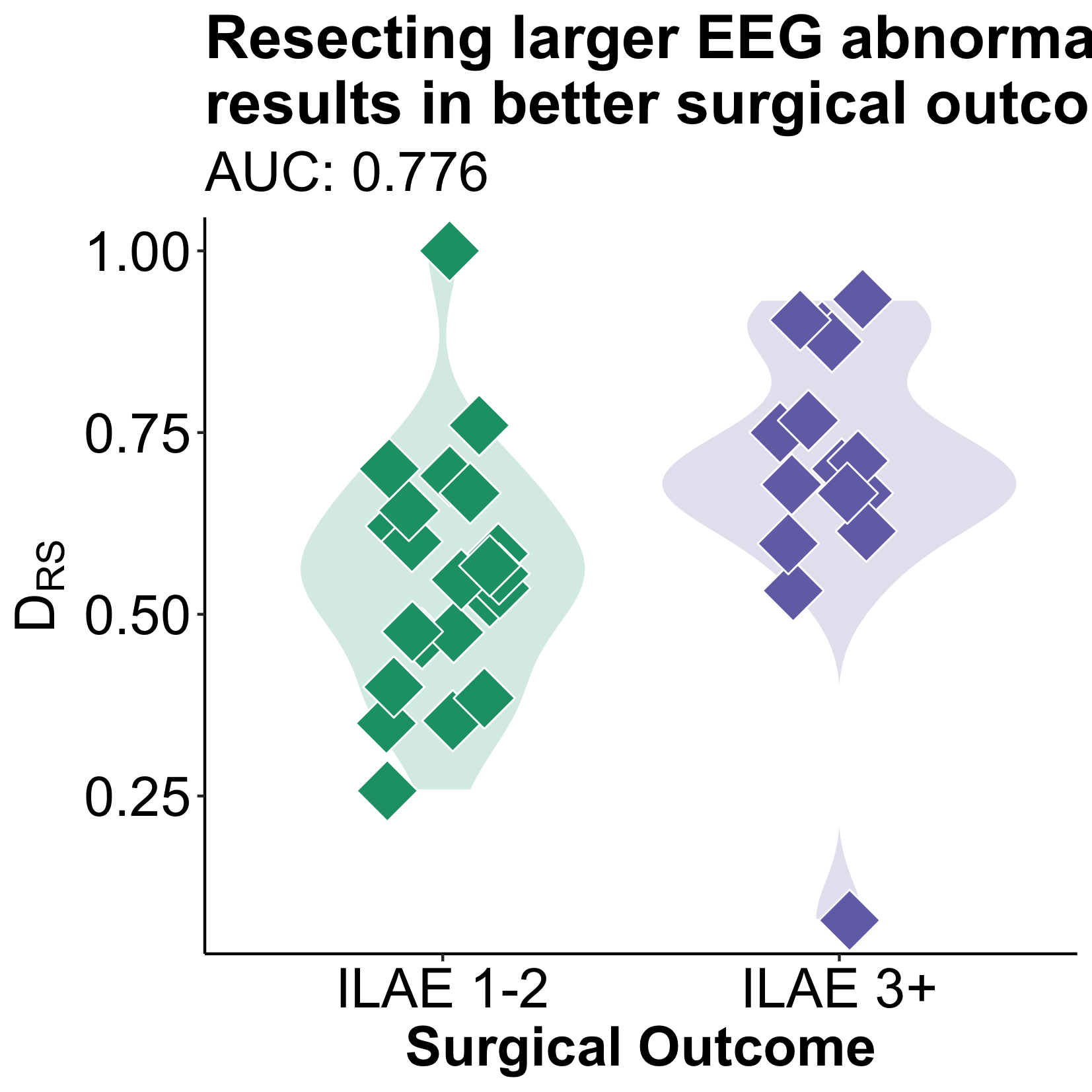

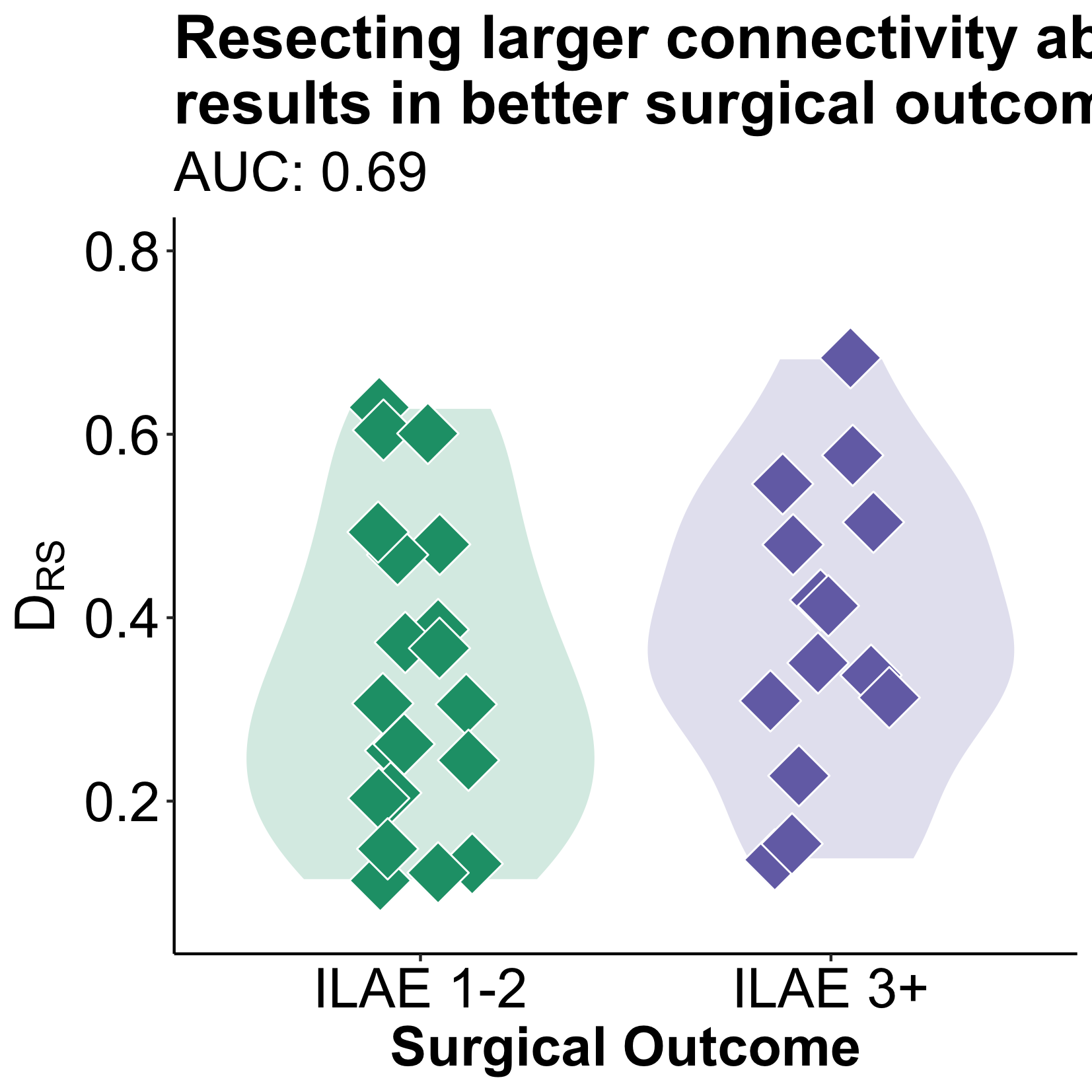


**Supplementary Figure 6: Patients with poor post-surgical outcomes had greater iEEG D_RS_ values (left) and larger connectivity D_RS_ values (right), at a regional resection threshold of 50%.**

**Supplementary Analysis 4 - Alternative Case Studies**

In the main manuscript, we presented a case study involving a patient who had the largest connectivity abnormalities resected and was seizure free. To provide more information on the value of this approach, we present four additional cases in Supplementary Figure 7 below. All four of these patients continued to have seizures post-surgery. In the first three cases (panels A, B and C), peak connectivity abnormalities were not implanted with iEEG or resected and could have been targeted for a potentially improved outcome. Interestingly in each case, regions in the same lobe as these peak abnormalities were implanted, suggesting that this lobe was being considered as a potential resection site.

For balance, we present a final case (panel D), in which our approach would have suggested a good post-surgical outcome since the largest connectivity abnormalities were resected. This highlights both a) the importance of the complementary iEEG information (high DRS suggesting most abnormal iEEG abnormalities were spared) and b) that our approach is not perfect.


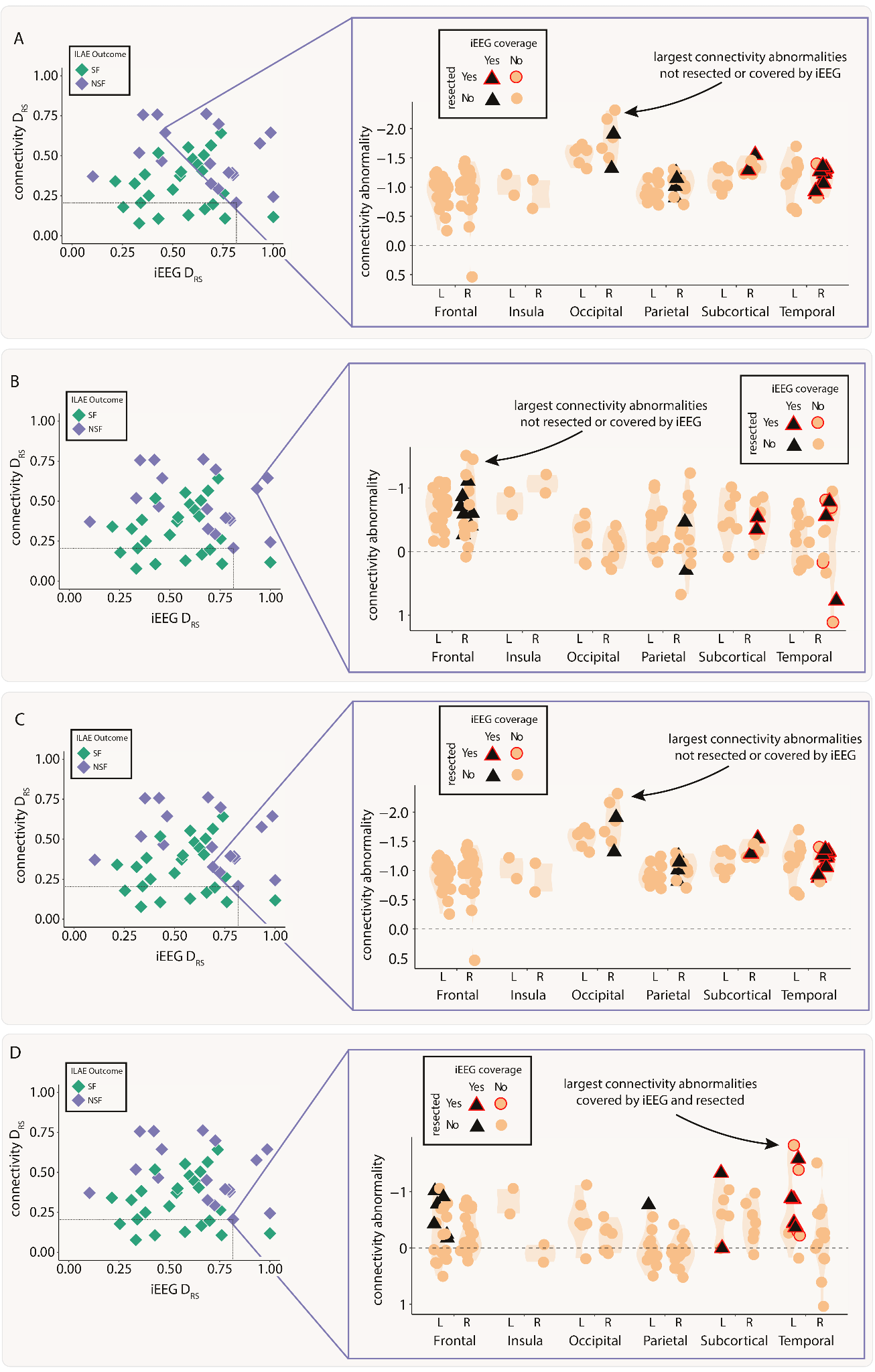


**Supplementary Figure 7: Alternative case studies for patients who were not seizure-free following surgery.**

**Supplementary Analysis 5 - Resection of Seizure Onset**

We investigated if similar results as presented in the main manuscript could be achieved by evaluating the seizure onset zone (SOZ). We classified a ROI as being SOZ if it contained at least one iEEG channel clinically marked as being SOZ. We then computed both the dice similarity between resected regions and SOZ regions and proportion of SOZ regions resected separately for each patient.

We found no significant difference in dice similarity in between good and poor outcome patients (p=0.48; Supplementary Figure 8, left panel). Similarly, we found no significant difference in proportion of SOZ regions resected between good and poor outcome patients (p=0.66; Supplementary Figure 8, right panel). This suggests that simply resecting SOZ regions does not lead to significantly better outcomes, as compared to resecting dMRI/iEEG abnormalities as presented in the main manuscript.


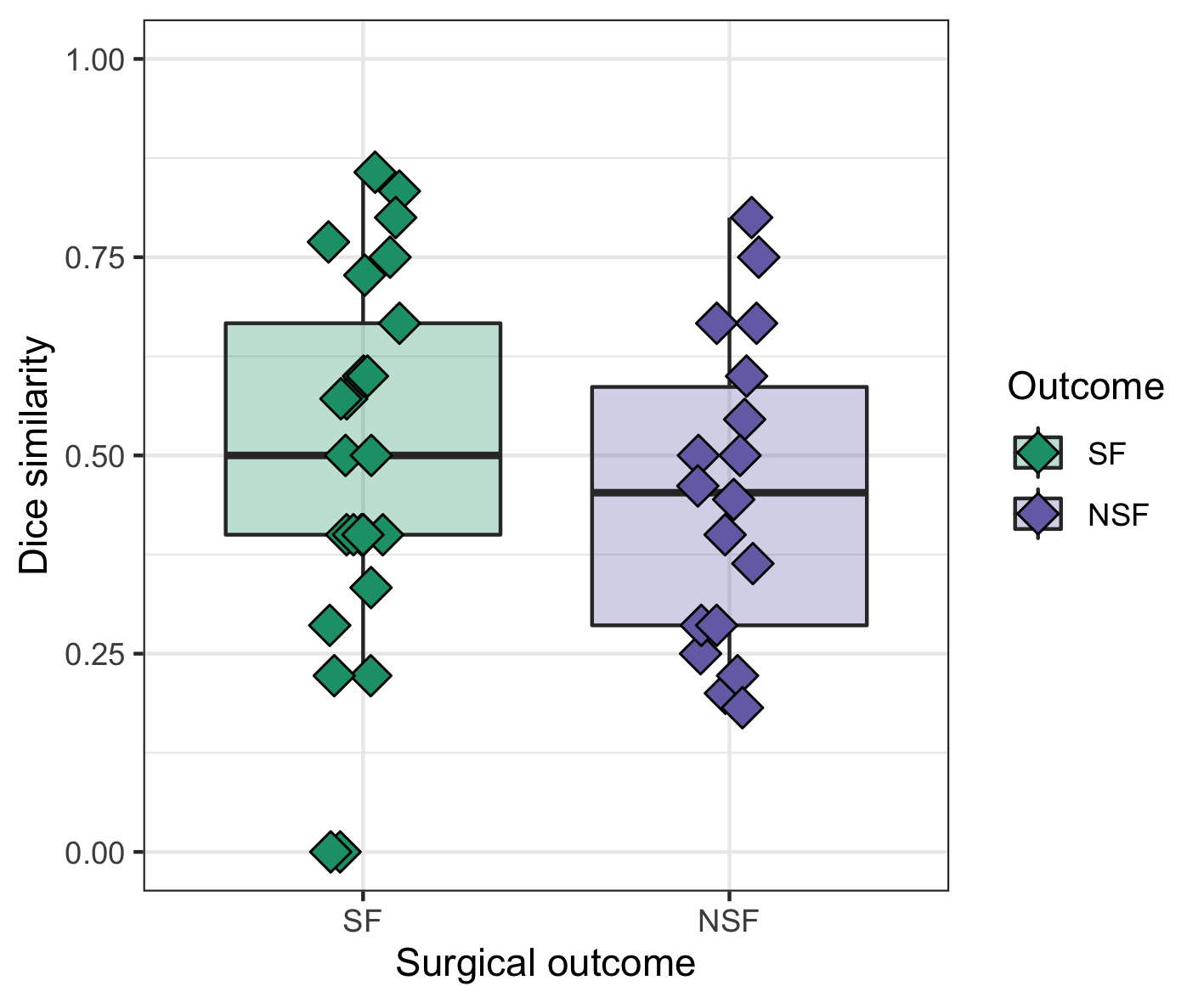

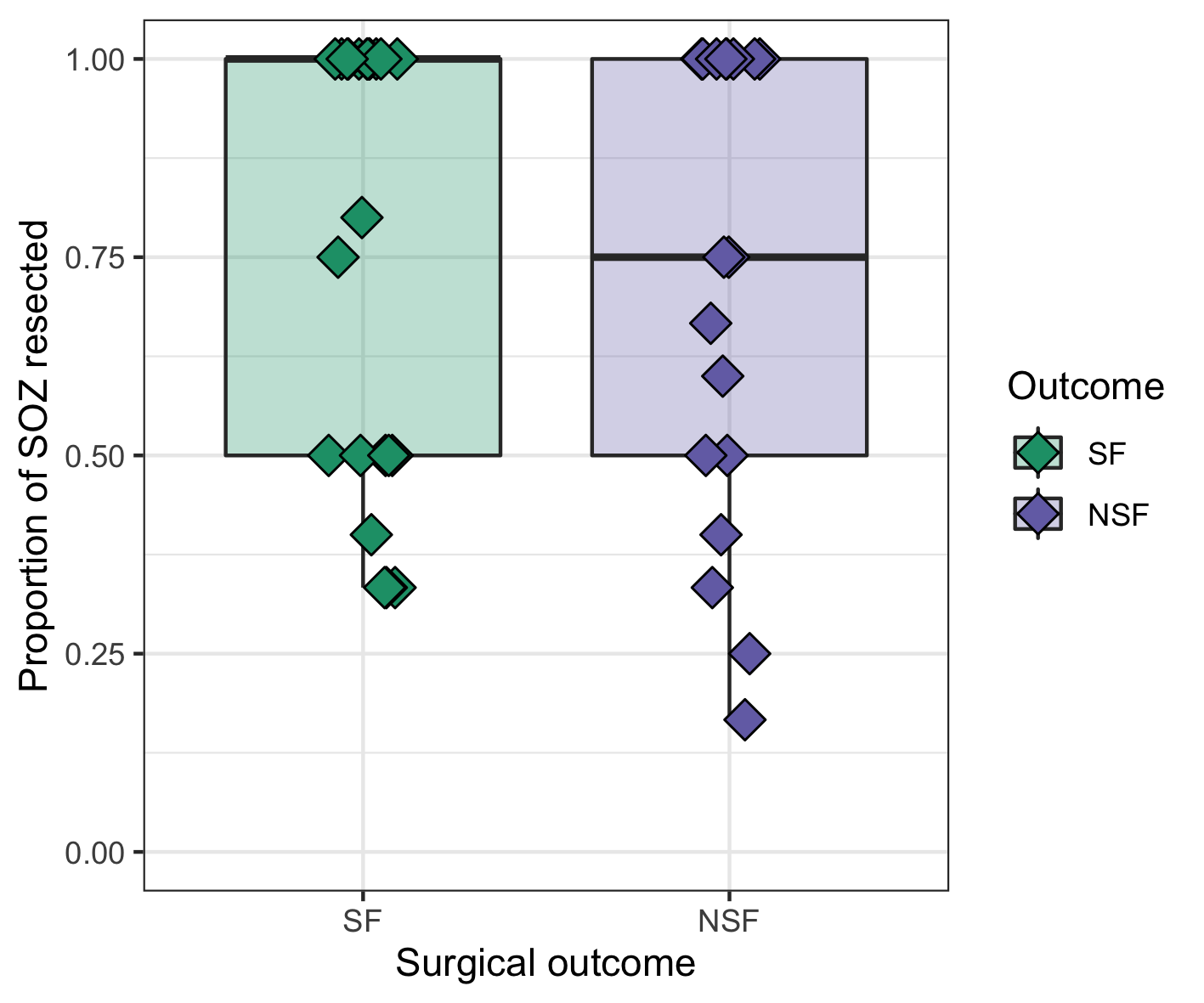


**Supplementary Figure 8: Resection of seizure onset regions does not distinguish outcome groups.**

Next, we replicated the analysis in Figure 4 of the manuscript, using distinguishability between seizure onset and not seizure onset (D_Onset-NotOnset_) instead of distinguishability between resected and spared regions(D_RS_). This is shown in Supplementary Figure 9..


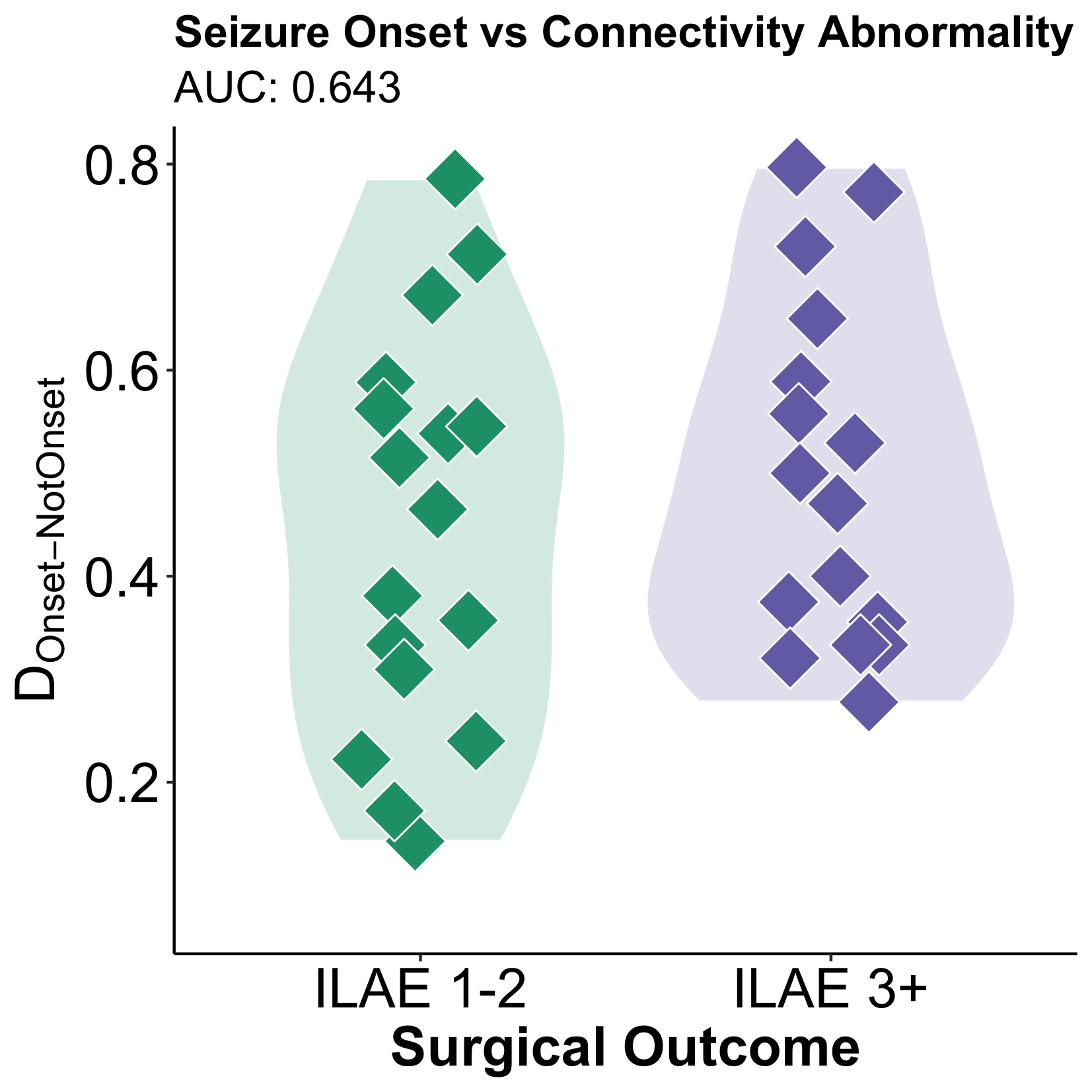

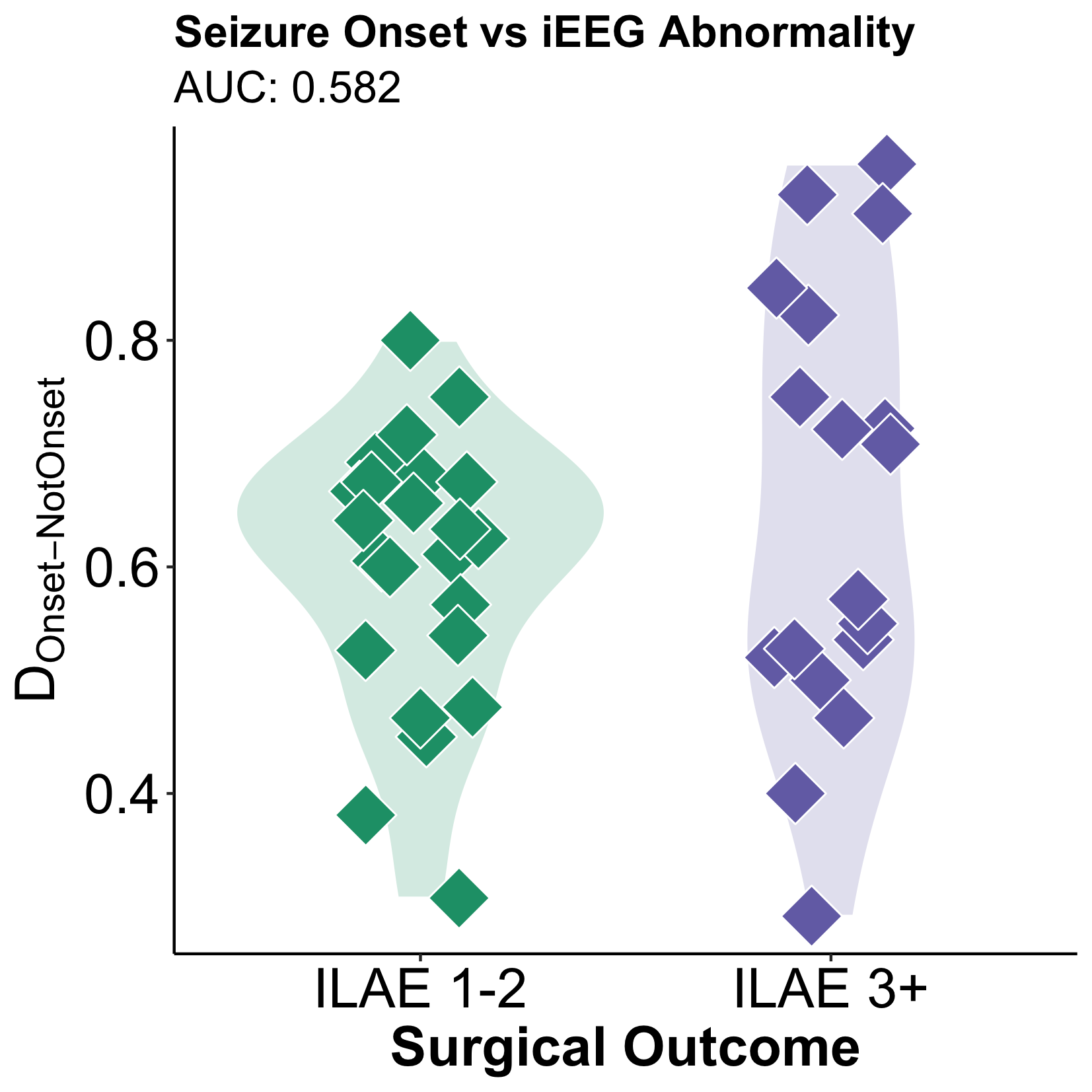


**Supplementary Figure 9**: Distinguishability between seizure onset and not-seizure onset (D_Onset-NotOnset_) regional abnormalities.

This analysis quantifies whether abnormalities were larger in regions marked as being SOZ, compared to those being marked as not SOZ (but still implanted with iEEG electrodes). A D_Onset-NotOnset_ value close to 0 indicates that abnormalities were larger in SOZ regions and smaller in not-SOZ regions. Vice versa, A D_Onset-NotOnset_ value close to 1 indicates that abnormalities were smaller in SOZ regions and greater in not-SOZ regions.

D_Onset-NotOnset_ values did not discriminate between surgical outcome groups (Wilcoxon rank-sum tests: iEEG p=0.38; Connectivity p=0.12).

**Supplementary Analysis 6 - MRI-positive and MRI-negative Subgroup Analysis**

We repeated the main analyses of the manuscript separately in the subsets of MRI-negative patients (n=20) and MRI-positive patients (n=23). The results are given below and have been added as a supplementary analysis. Note that the smaller sample sizes will result in less statistical power (i.e. larger p-values for the same effect size) compared to the full cohort. Overall, results agree with the conclusions of the full cohort presented in the main manuscript.

**MRI-Negative**

In the subset of 20 MRI-negative patients, the SVM successfully separated the resected and spared abnormalities in 15 patients. Of these 15 patients, resection of maximal abnormalities was significantly associated with better post-surgical outcomes (Supplementary Figure 10; accuracy = 0.87, sensitivity = 0.89, specificity = 0.83, odds ratio = 40, p=0.02).


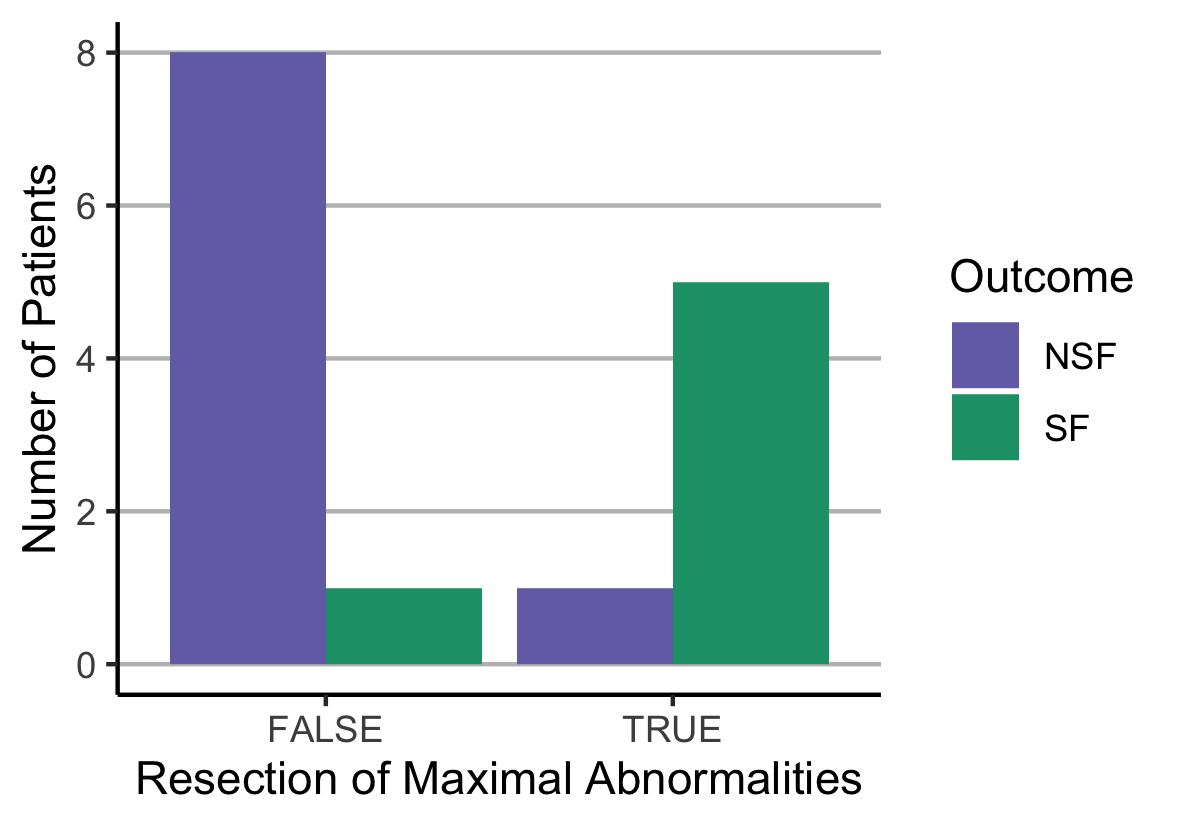


**Supplementary Figure 10: MRI-negative patients with resection of maximal abnormalities were significantly more likely to be seizure-free.**

In the subset of 20 MRI-negative patients, iEEG D_RS_ values were significantly greater in poor outcome (NSF) patients (Supplementary Figure 11, AUC = 0.76, p=0.03). Similarly, connectivity D_RS_ values were greater (almost significantly) in poor outcome patients (Supplementary Figure 11, AUC = 0.71, p=0.06).


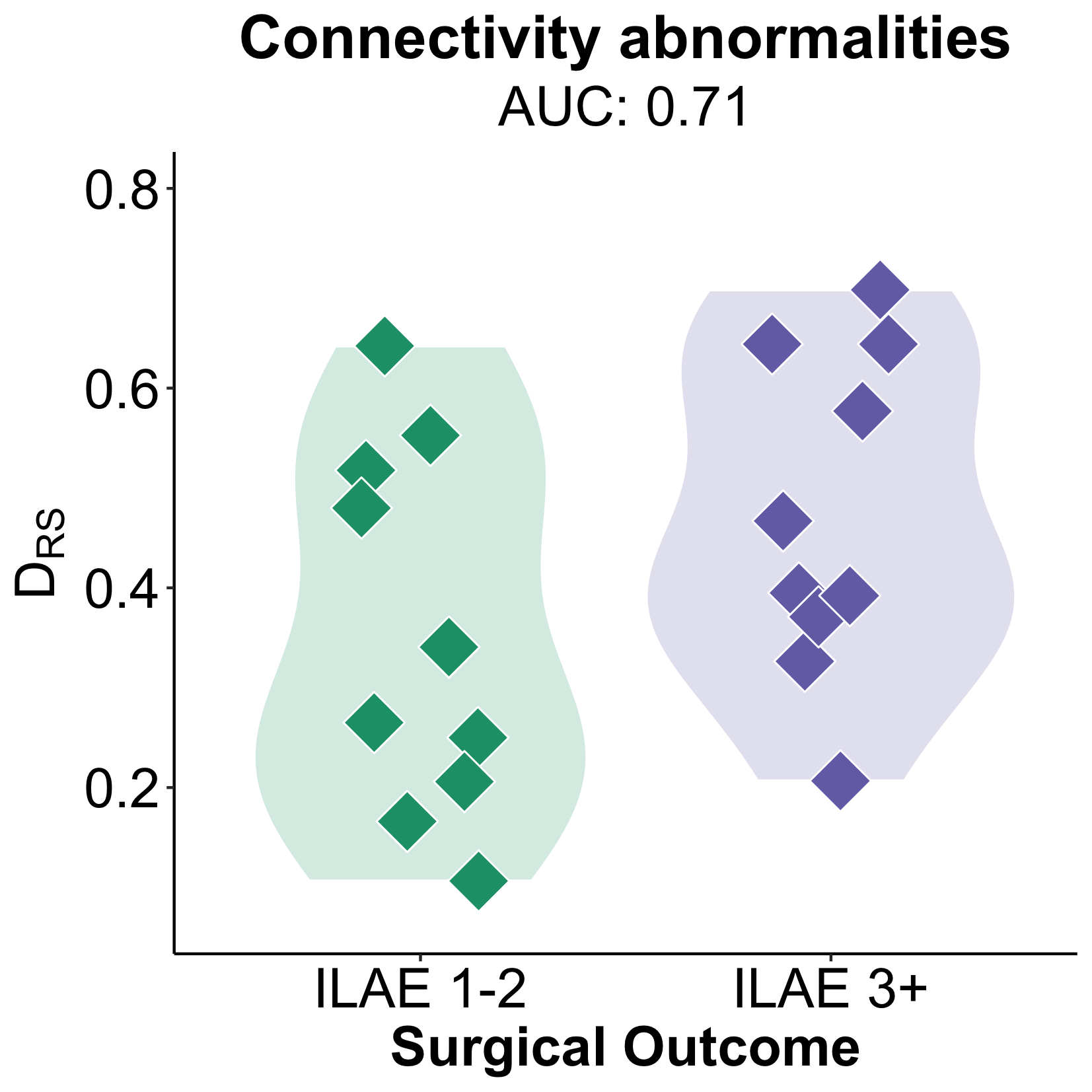

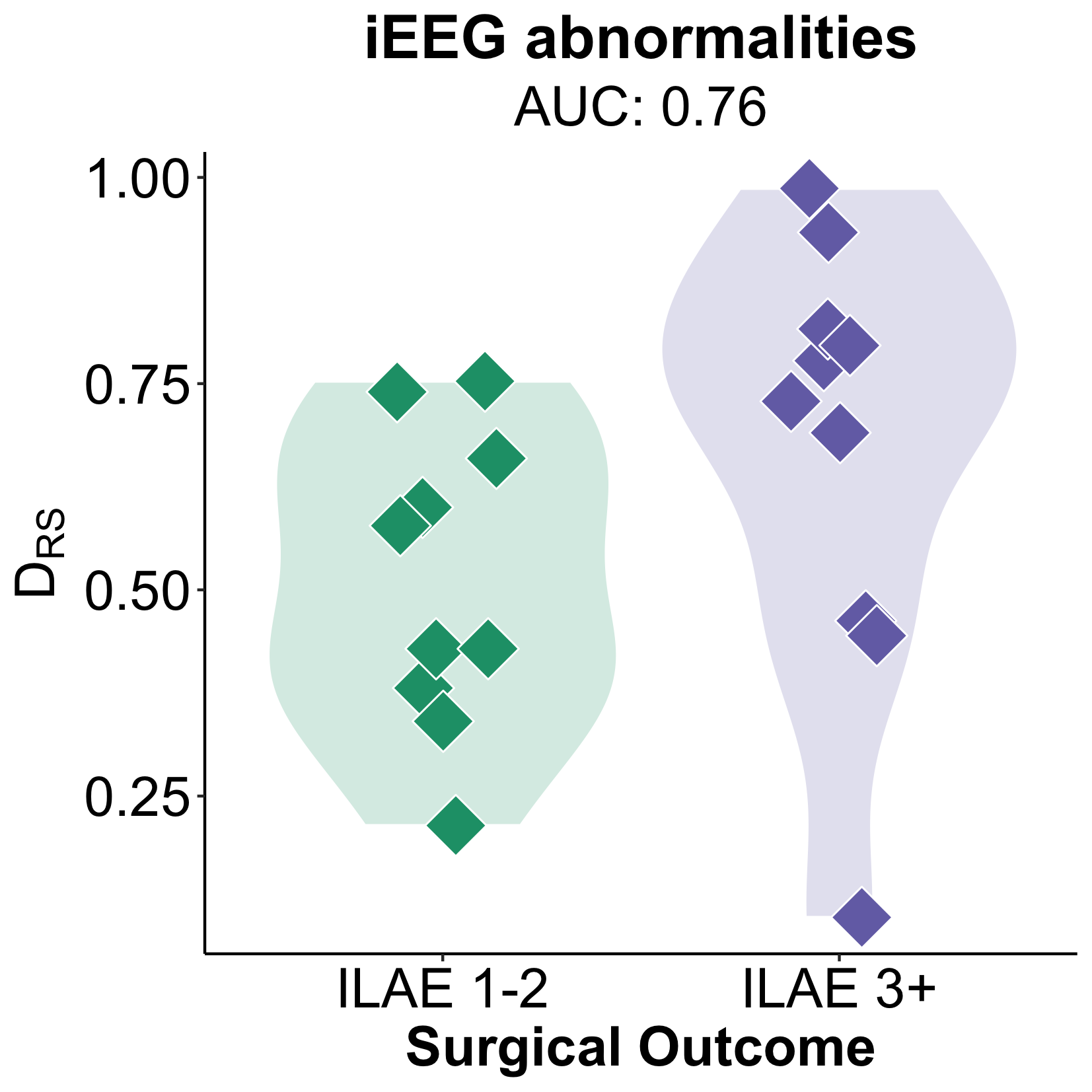


**Supplementary Figure 11: Distinguishability between resected and spared (D_RS_) regional abnormalities in MRI-negative patients.**

**MRI-Positive**

In the subset of 23 MRI-positive patients, the SVM successfully separated the resected and spared abnormalities in 13 patients. Of these 13 patients, resection of maximal abnormalities was not significantly associated with better post-surgical outcomes (Supplementary Figure 12, accuracy = 0.69, sensitivity = 0.57, specificity = 0.83, odds ratio = 6.67, p=0.36). However, the trend for resection of maximal abnormalities to occur more frequently in SF patients was still evident.


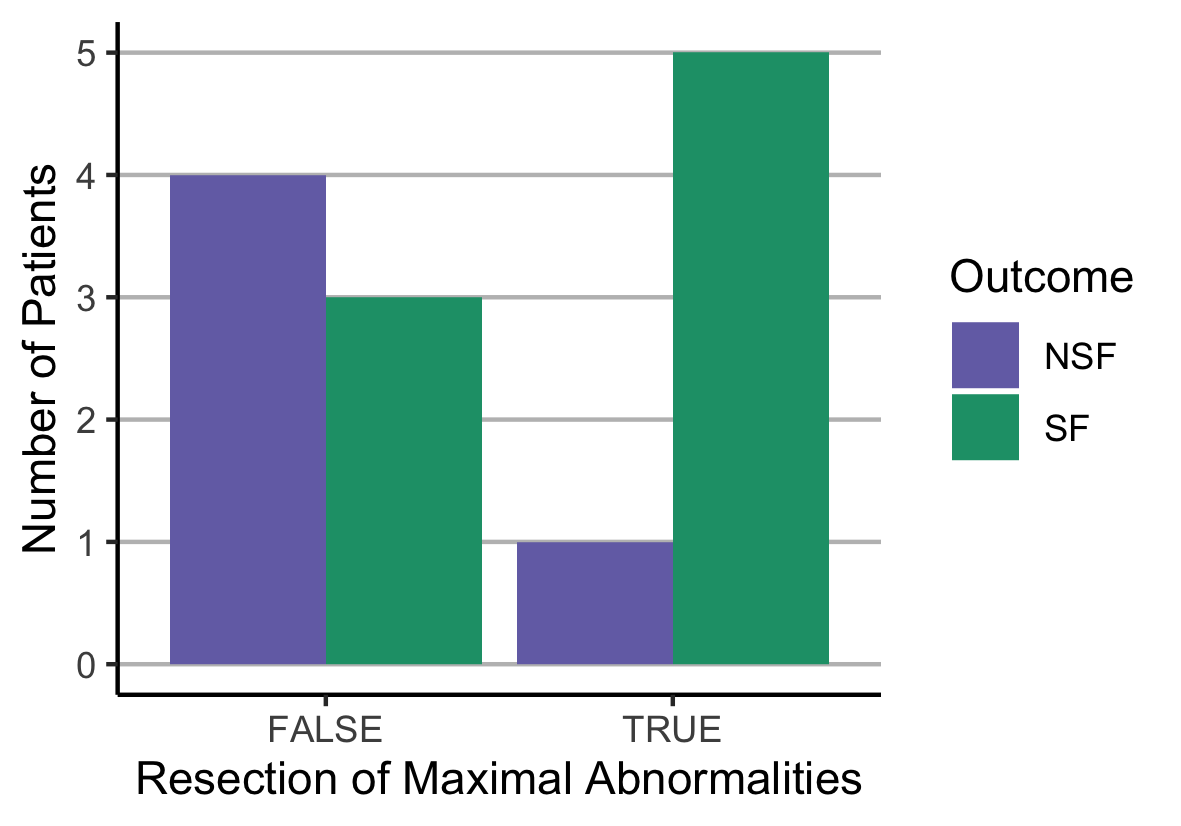


**Supplementary Figure 12: Association between resection of maximal abnormalities and seizure freedom in MRI-positive patients.**

In the subset of 23 MRI-negative patients, iEEG D_RS_ values were not significantly greater in poor outcome (NSF) patients (Supplementary Figure 13, AUC = 0.61, p=0.21). However, connectivity D_RS_ values were significantly greater in poor outcome patients (Supplementary Figure 13, AUC = 0.78, p=0.01).


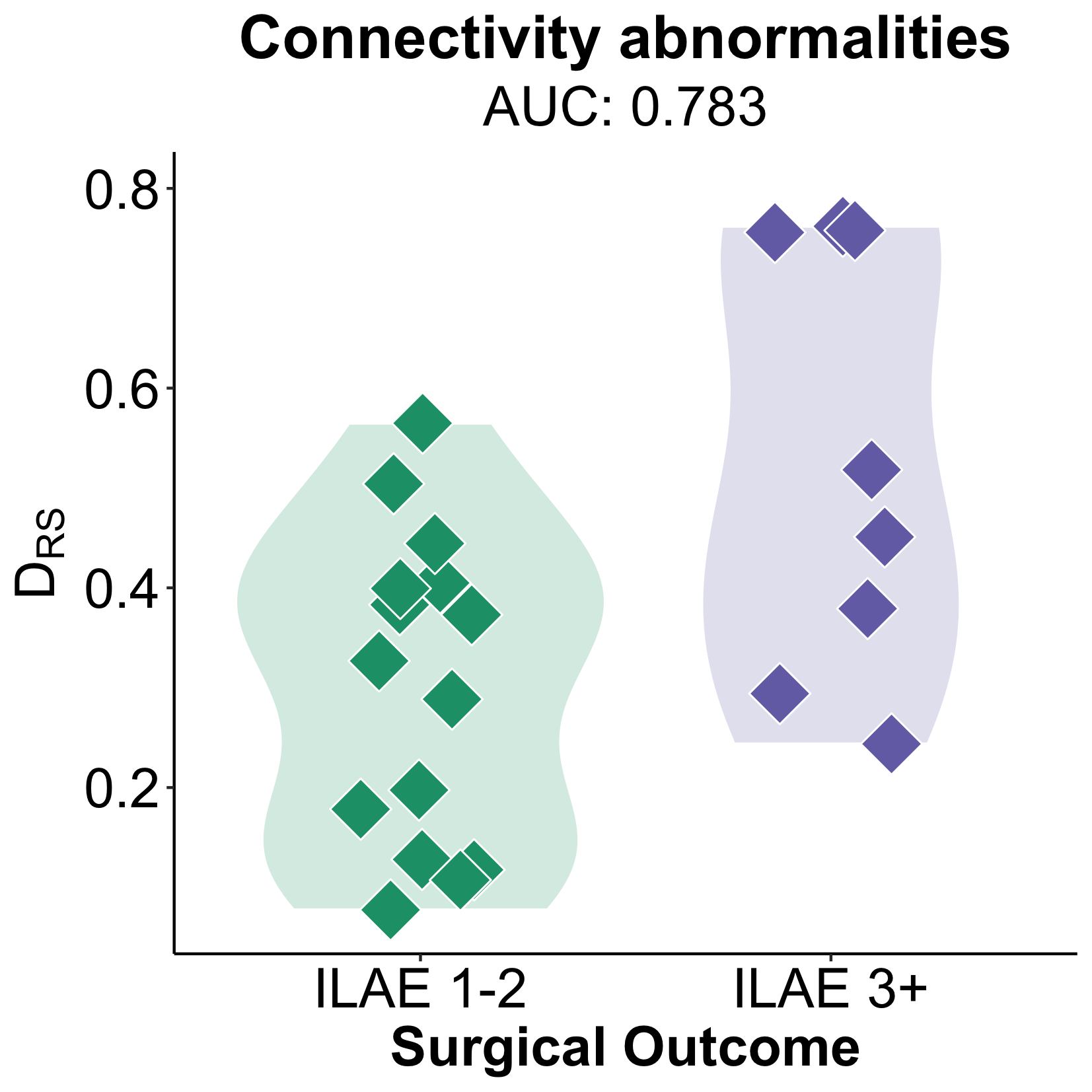

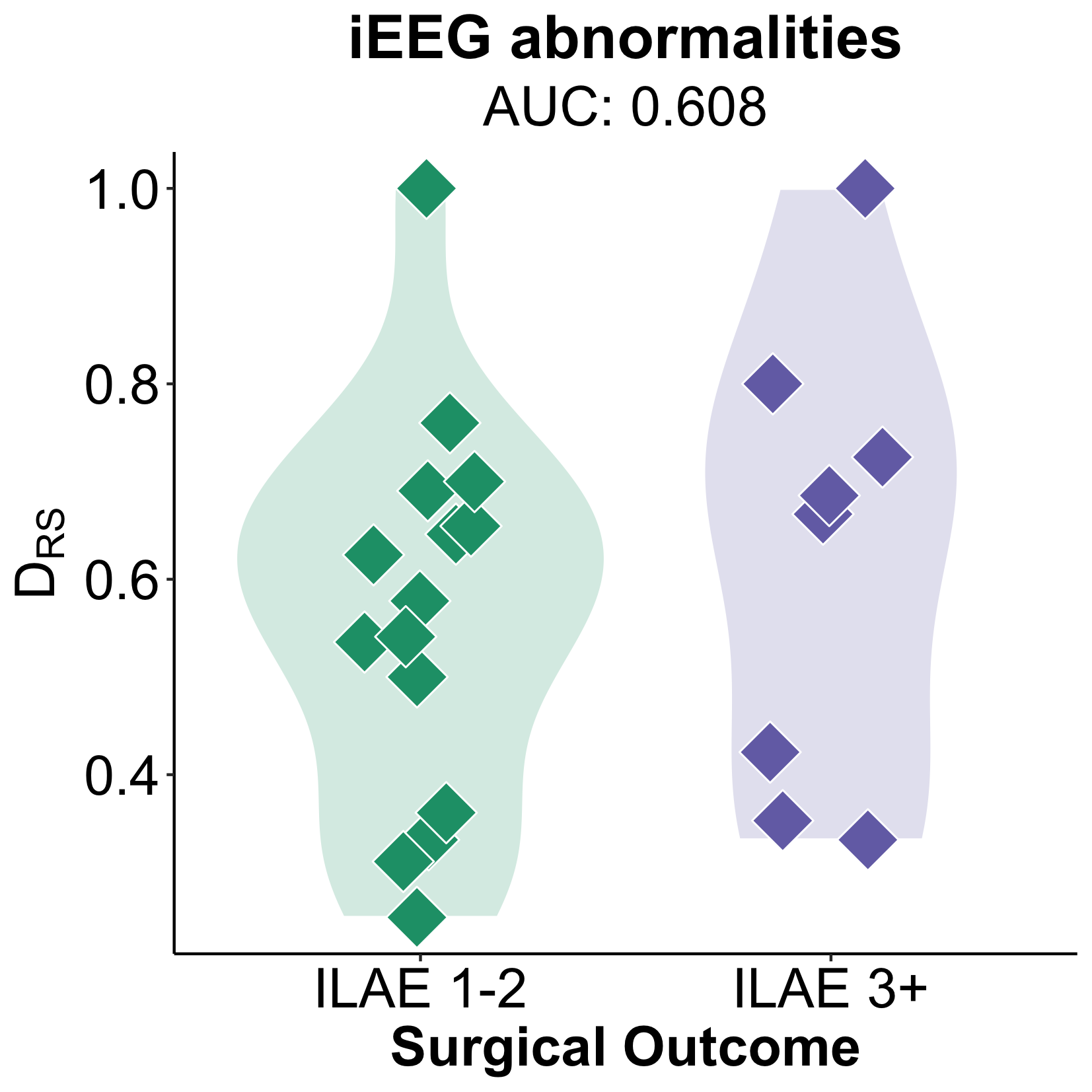


**Supplementary Figure 13: Distinguishability between resected and spared (D_RS_) regional abnormalities in MRI-positive patients.**

**Supplementary Analysis 7 - Further Example Patients (eTLE)**

In the main manuscript, we presented Patients 1 and 2. These two patients were examples of seizure-free and not seizure-free patients and both were temporal cases. Here we present an additional two examples of extra-temporal cases. Patient 3 had a left frontal resection and was seizure-free following surgery (ILAE 2). Patient 4 had a right parietal resection and was not seizure-free following surgery (ILAE 4).

Of the regions implanted with iEEG in Patient 3, the largest connectivity abnormalities occurred in the left frontal lobe and were resected. Additionally (with the exception of one temporal region), the largest iEEG abnormalities were typically resected (Supplementary Figure 14 - Panel A). This patient did not have seizures following surgery.

Of the regions implanted with iEEG in Patient 4, the largest connectivity abnormalities occurred in frontal and parietal regions and were not resected. Additionally, the largest iEEG abnormalities occurred in parietal regions were not resected (Supplementary Figure 14 - Panel B). This patient continued to have seizures following surgery.

**
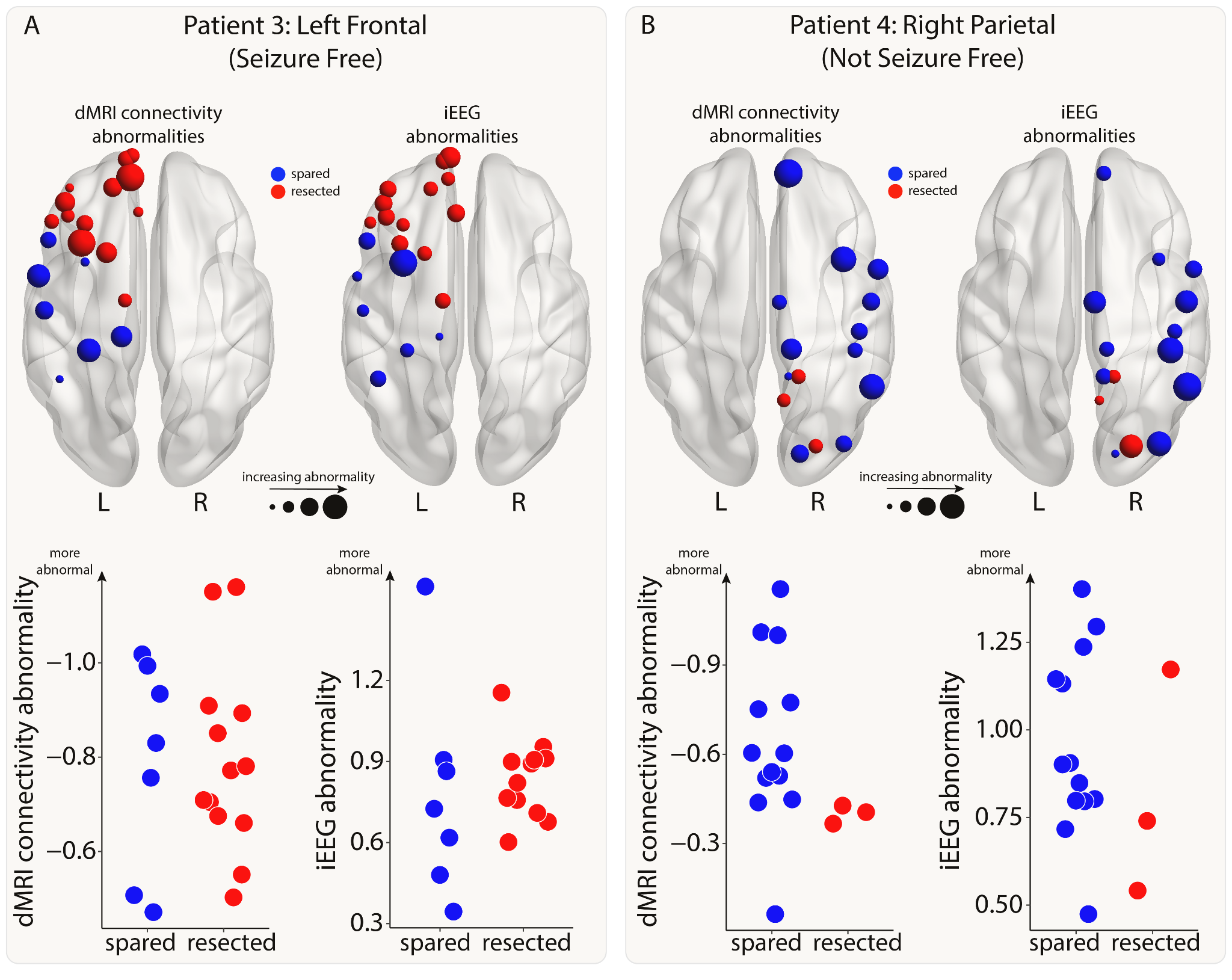
**

**Supplementary Figure 14: Connectivity and iEEG abnormalities in example eTLE patients.**
